# The British Farmers' Hospital

**Published:** 1919-05-17

**Authors:** 


### The British Farmers' Hospital.

Miss Madel L. Stokes, matron, writing from the British Farmers' Hospital, Rue Raymond Blychaerts XL, Brussels, asks us to publish the following "collective letter," which, it will be observed, is unsigned :

The matron and nursing staff of the British Farmers' Hospital in Brussels are indignant, and wish to protest against an article which appeared in The Hospital number of April 26, 1919, entitled "The River Past and God Forgotten." In it there is a general misrepresentation of the facts, which we will not discuss, because they come outside our province. The only matter we wish to take up is the allegations which concern the nursing staff. The salaries and demobilisation bonus are overdue, through no fault of the British Farmers Committee, but owing, we think, apparently to lack of cohesion between the London and Boulogne offices of the British Red Cross Society. However, knowing that the Joint War Committee have to deal with the demobilisation of hundreds of personnel, we are willing enough to wait patiently. The members of the British Farmers' Committee have been doing their best to get out of this difficulty as soon as possible. The nursing staff has always received the utmost consideration at their hands, and is proud to work for them. They are quite happy and contented in Brussels. The delay in the payment of salaries and demobilisation bonus has been a little inconvenient, but not one formal complaint has been made. The nursing sisters are not penniless; each one had received regularly the proportion of her salary which she had asked might be paid to her while abroad. The sisters are so sure that their interests are safe in the hands of the British Farmers' Committee that no one wished to make any fuss about the matter.

[The subjects raised are dealt with in this issue under the heading, "The River Past, God Forgotten, and the Ferryman Punished." See p. 160.]

